# Gut as a Target of Ochratoxin A: Toxicological Insights and the Role of Microbiota

**DOI:** 10.3390/ijms26199438

**Published:** 2025-09-26

**Authors:** Magdalena Więckowska, Rafał Szelenberger, Tomasz Poplawski, Michal Bijak, Leslaw Gorniak, Maksymilian Stela, Natalia Cichon

**Affiliations:** 1 Biohazard Prevention Centre, Faculty of Biology and Environmental Protection, University of Lodz, Pomorska 141/143, 90-236 Lodz, Poland; magdalena.wieckowska@biol.uni.lodz.pl (M.W.); rafal.szelenberger@biol.uni.lodz.pl (R.S.); michal.bijak@biol.uni.lodz.pl (M.B.); leslaw.gorniak@biol.uni.lodz.pl (L.G.); maksymilian.stela@biol.uni.lodz.pl (M.S.); 2 Department of Microbiology and Pharmaceutical Biochemistry, Medical University of Lodz, Mazowiecka 5, 92-215 Lodz, Poland; tomasz.poplawski@umed.lodz.pl

**Keywords:** ochratoxin A, microbiome, intestine, biodetoxification, dysbiosis

## Abstract

Ochratoxin A (OTA) is a widespread foodborne mycotoxin that poses significant risks to both human and animal health. Upon ingestion, the gastrointestinal tract (GIT) becomes the main site of exposure, where OTA interacts directly with the intestinal epithelium and resident microbiota. Research indicates that OTA disrupts the integrity of the intestinal barrier and alters its permeability. Moreover, OTA undergoes transport and partial metabolism within the intestine before being excreted. Detoxification pathways for OTA include enzymatic degradation and adsorption by microorganisms. Notably, OTA has profound toxic effects on the gut ecosystem; it can alter the relative abundance of bacterial taxa by reducing beneficial populations and promoting opportunistic or pathogenic strains. These changes contribute to an imbalance in the microbiota, impairing host metabolic and immune functions. This dysbiosis is characterized by disrupted microbial homeostasis and impaired communication between the host and its gut microbiome. This review highlights the dual role of the intestine as both a target and a modulator of OTA toxicity. It emphasizes the importance of gut microbiota in mediating the toxicological outcomes of OTA and explores microbiome-based strategies as potential avenues for detoxification.

## 1. Introduction

Ochratoxin A (OTA) is the most toxic among ochratoxins synthesized by *Aspergillus* and *Penicillium* fungi, depending on the climate [1]. In temperate and cold regions, *Penicillium verrucosum* is the primary source of OTA, whereas in warm and tropical areas, production is mainly associated with *Aspergillus ochraceus*. Other *Aspergillus* species, including *A. niger* and *A. carbonarius*, also contribute significantly, particularly in commodities such as coffee and maize. The efficiency of OTA biosynthesis depends strongly on environmental conditions, especially temperature and water activity. Generally, higher water activity and moderate temperatures favor toxin production, while xerotolerant species such as *A. niger* can maintain growth and OTA synthesis under more extreme conditions [2]. These differences highlight the ecological diversity of OTA-producing fungi and the importance of environmental factors in determining contamination risk.

OTA has been found to possess several harmful effects, including embryotoxic, teratogenic, genotoxic, neurotoxic, nephrotoxic, and immunosuppressive properties [3]. The International Agency for Research on Cancer has classified OTA as a potential human carcinogen in group 2B [4], emphasizing its multifaceted and detrimental nature. For this reason, it poses a huge risk associated with its consumption due to its presence in many foods, beverages [5,6,7,8,9,10,11,12], and animal-derived products [13,14,15,16,17,18] due to its presence in animal feed, and its ability to accumulate in tissues [19,20,21,22,23,24]. In wildlife, OTA has been primarily detected in the kidney and liver tissues of wild boars, highlighting the potential for human exposure through the consumption of wild game meat [25]. Among processed meat products, an analysis of 172 Italian salamis revealed OTA in 22 samples, with three exceeding the national guidance value of 1 µg/kg for pork products. Notably, the majority (68.2%) included spicy salamis [16]. The occurrence of OTA has also been reported in spices and herbs, with 30% of spice and 11% of herb samples, at a mean concentration of 7.2 µg/kg and 7.0 µg/kg, respectively. Contamination included cloves, coriander, rosemary, sage, and oregano, suggesting their importance in dietary risk [8]. Additionally, OTA was detected in 5% of the analyzed nut and dried fruit samples [6].

The intestines that come into contact with OTA as a result of its extensive metabolism are subject to its toxic effects [26,27,28]. Additionally, when discussing the intestines as an organ, attention should also be given to the microbiome. Its definition refers to the set of genes of microorganisms found in the human body [29,30]. The human microbiome is mostly present in the gastrointestinal tract (GIT), and its important role is to influence the development, functioning, and homeostasis of the GIT, as well as its integration with the host’s immune and nervous systems [31].

The microbiota is present wherever contact with the external non-sterile environment exists and consists of various bacteria, archaea, fungi, protozoa, and viruses. Therefore, they inhabit various organs of the body, including the intestines, which impacts their characteristic feature [32]. This is related to their ability to adapt to the conditions prevailing in the human body, and therefore to factors such as temperature, pH, oxygen concentration, pressure, osmolarity, and source of nutrients. In addition, the abundance and diversity of the microbiota are influenced by other internal factors such as genetics, ethnicity, gender, and age, as well as external factors including diet, lifestyle, medication, climate, and seasonality [29].

The gut microbiota is the densest and most diverse. Most commensal bacteria inhabit the colon, while the small intestine is less populated. The intestines are inhabited by numerous types of bacteria, such as *Firmicutes* and *Bacteroides*, which are a significant advantage over others [29]. Gut bacteria are crucial for regulating digestion, since they play a key role in processing nutrients and metabolites, resulting in increased energy harvesting and metabolic efficiency. Some bacteria play an immunological role against pathogens. However, when the bacterial flora is disrupted, intestinal permeability increases, which ultimately contributes to the invasion and colonization of these pathogens. Increased intestinal permeability consequently leads to the production of dysregulated metabolites and the penetration of microbial products, disrupting the proper functioning of the gut microbiome and contributing to abnormal immune-inflammatory responses [29].

Due to the exposure of the GIT to OTA, we decided to examine this issue, given the numerous studies indicating the toxic effects of OTA on the intestines and its impact on the microbiome, taking into account its role in biodetoxification and dysbiosis.

## 2. Intestines and Ochratoxin A Pharmacokinetics

Upon ingestion, OTA is absorbed through the GIT into the systemic circulation, where it forms complexes with serum proteins, predominantly albumin [1]. The toxin is effectively transported among tissues as a result of enterohepatic recirculation, a process that entails the reabsorption of OTA from the intestines back into the bloodstream. Furthermore, OTA is reabsorbed in both the proximal and distal tubular regions of the kidneys. Consequently, OTA accumulates primarily in the bloodstream and organs such as the kidneys and liver, which are also the principal sites of its biotransformation [1]. After biotransformation, OTA and/or OTA metabolites are excreted through milk, urine, and feces [1,19]. Vila-Donat et al. conducted an in vivo study on Wistar rats exposed to OTA to assess the presence of the toxin in fecal samples. As reported, after four weeks, the concentration of OTA in dry feces reached 1729 ± 712 µg/kg in males and 933 ± 512 µg/kg in females [33]. Zhu et al. investigated the toxicokinetics of OTA in lactating sows after a single oral dose of 500 µg/kg. The toxin was excreted through feces and urine over 120 h, accounting for nearly 19% of the administered dose. Importantly, OTA residues were also detected in milk [34]. Geographical and individual variations in OTA levels in human milk have been linked to dietary patterns. A study on 80 Norwegian women reported that 21% of milk samples contained detectable OTA concentrations (10–182 ng/L). Higher toxic occurrence was associated with frequent consumption of liver-based products, cakes, and juices. Additionally, cereals, processed meats, and cheese were also identified as potential contributors [35]. Similar findings were reported by Breitholtz-Emanuelsson et al., in which the researchers observed OTA presence in 14% of cow’s milk and 58% of human milk [14].

Mycotoxins significantly affect intestinal xenobiotic transport and metabolism through their interactions with biotransformation enzymes and efflux proteins. Tuntiteerawit et al. demonstrated that inhibition of breast cancer resistance protein (BCRP) in Caco-2 cells markedly reduced Aflatoxin B1 (AFB1) efflux and increased intracellular accumulation [36]. Similarly, deoxynivalenol (DOX) and fumonisins (FBs) have been shown to influence xenobiotic disposition in poultry by altering the expression of cytochrome P450 (CYP450) enzymes and multidrug resistance transporters. In particular, FB-contaminated feed significantly upregulated CYP1A4 and Multidrug Resistance Protein 1 (MDR1) expressions in the jejunum, and was associated with reduced oral bioavailability of enrofloxacin, a known MDR1 substrate [37]. Similarly, as demonstrated by Schrickx et al. [38], in Caco-2 cell monolayers, OTA was preferentially secreted to the luminal side in a concentration-dependent manner. Furthermore, inhibition of both ATP-dependent transporters multidrug resistance-associated protein 2 (MRP2) by MK571 and BCRP by Ko143 and by GF120918, as well as cyclosporine A, reduced secretion and increased absorption, indicating their involvement in OTA efflux [38]. The findings of these studies emphasize the critical role of the intestines in systemic bioavailability of OTA and its toxicological outcomes.

## 3. Biodetoxification of OTA

The presence of mycotoxins in food can lead not only to significant economic losses due to the necessity of disposing of contaminated products, but also to poisoning and serious risks to human health and life. Therefore, it is essential to develop methodologies that enable the detoxification of mycotoxins in both the agricultural and medical sectors [39].

Detoxification methods used for food decontamination can be categorized into physical, chemical, and biological approaches. Physical methods—such as sorting, processing, heat treatments, and irradiation—often fail to achieve high efficiency and, consequently, may not remove mycotoxins to the required level, rendering them impractical in certain cases [40,41]. On the other hand, chemical detoxification can lead to secondary contamination due to the presence of chemical residues in the food and feed. Moreover, the use of chemical agents may negatively affect the sensory attributes of food, including changes in odor, taste, appearance, and even loss of nutritional value [42,43]. A third approach, which has attracted significant scientific interest, is biological detoxification. This method is considered both cost-effective and environmentally friendly, offering a promising strategy for the removal of mycotoxins from food [39,43]. This approach involves the use of microorganisms such as yeasts, bacteria, and fungi, as well as their enzymes and metabolites, to bind and degrade mycotoxins [39,42]. Importantly, only microorganisms recognized as safe (non-pathogenic) and those that do not alter the sensory properties may be used [42].

Studies aimed at developing methods for the biological detoxification of mycotoxins have demonstrated that the reduction in their concentration in both solid and liquid food products can be achieved through adsorption, chemical degradation, and biodegradation, mediated by biologically active compounds produced by bacterial strains with mycotoxin-degrading capabilities [43,44].

Biodetoxification of OTA is primarily based on two distinct pathways. One of the mechanisms used for its degradation is the use of enzymes, such as carboxypeptidase (CP). Functioning as a hydrolase, this enzyme specifically targets and cleaves the amide bond in the OTA molecule, resulting in the formation of smaller and much less toxic compounds, such as ochratoxin α (OTα), which is regarded as non-toxic or exhibits at least a 500-fold lower toxicity compared to OTA, and L-β-phenylalanine [40,45]. This mechanism is crucial due to the significant limitation in the generation of harmful intermediate products (Table 1) [40].

The first report indicating the possibility of OTA degradation by enzymes was published in 1969 by Pitout et al. [46]. In the study conducted by Stander et al., 23 commercially available enzymes, especially lipases, were evaluated for their capacity to degrade OTA. Among them, only the lipase derived from *Aspergillus niger* exhibited a high hydrolytic efficiency, as confirmed by chromatographic analyses [47].

A similar study was performed by Abrunhosa et al., in which the authors evaluated whether the commercially available enzymes, especially proteases, possess the ability to hydrolyze OTA. Results showed that Protease A (*A. niger*), Prolyve PAC (*A. niger*), and pancreatin (porcine pancreas) are able to cleave OTA. The conversion from OTA to OTα for tested enzymes was 87.3% (after 25 h, pH 7.5, 37 °C), 3% (after 25 h, pH 3, 37 °C; at pH 7.5, hydrolytic activity was not detected), and 43.3% (after 25 h, pH 7.5, 37 °C), respectively. Moreover, the authors prepared an enzymatic extract (Ancex) from *A. niger* MUM 03.58, thus showing greater hydrolytic activity compared to CPA and resulting in 99.8% of OTA conversion into OTα in the same conditions [48]. *A. niger* is also a source of ochratoxinase—an enzyme belonging to the amidase family—which, in studies conducted by Dobritzsch et al., exhibited nearly 600-fold higher activity than CPA in the hydrolysis of OTA [49].

In the study performed by Chang et al. *Bacillus amyloliquefaciens* ASAG1 in liquid cultures and cell-free extracts showed 98.5% degradation of OTA in 24 h, and 99.7% degradation after 72 h. Furthermore, the authors cloned the CP from *B. amyloliquefaciens* and evaluated its role in the OTA degradation. The results showed that the addition of a purified CP led to a 72% reduction in OTA concentration [50]. Another microorganism exhibiting the ability to hydrolyze OTA is the *Acinetobacter* sp. neg1, ITEM 17016 strain, which was isolated from OTA-contaminated soil. The study revealed that incubation of OTA with ITEM 17016 strain resulted in its biotransformation from OTA into OTα, with over 70% rate after 144 h. Furthermore, the authors utilized the PJ15-1540 gene, which encodes CP, to assess the enzymatic activity in cell lysates. The results indicated that the coded protein was capable of converting approximately 33% of OTA into OTα [51]. According to Rodriguez et al., *Brevidobacterium casei* (RM101, DSM 20657, DSM 9657, DSM 20658), *B. linens* (DSM 20425), *B. iodinum* (DSM 20626), and *B. epidermidis* (DSM 20660) were capable of degrading all OTA in culture medium (concentration 40 µg/L), reaching 100% of efficacy [52]. High degradation efficacy was also shown for *Pediococcus parvulus* isolated from Douro wines, reaching 50% and 90% after 6 and 19 h, respectively [53].

**Table 1:** The summary of the microorganisms/enzymes able to degrade OTA with the production of OTα.

Microorganism	Reaction Conditions	Medium	OTA Concentration	The Percentage of OTA Degradation	Reference
*Yarrowia lipolytica* Y-2	Rotatory shaker 180 rpm, 28 °C, 20 h	PM Broth	1 µg/mL	97.2%	[54]
*Alcaligens faecalis* 0D-1	30 °C, 48 h	LB Medium	1 µg/mL 2 µg/mL 5 µg/mL	Up to 100% 22–64% 23–68%	[55]
*Lysobacter* sp. CW239	With agitation at 180 rpm, 30 °C, 24 h	LB Broth	30 µg/L	86.2%	[43]
*Bacillus amyloliquefaciens* ASAG1	31 °C, 10 h	No 4. Medium	1 µg/mL	98.5%	[50]
*Bifidobacterium bifidum* CECT 870T	37 °C, 24 h	MRS Medium	0.6 µg/mL	pH 3.5/pH 6.5 80.4/74.1	[56]
*Bf. breve* CECT 4839T				87.2/94.1	
*Lactobacillus bulgaricus* CECT 4005				73.9/96.4	
*Lb. casei* CECT 4040				81.4/88.5	
*Lb. casei* CECT 4045				87.1/88.5	
*Lb. johnsonii* CECT 289				76.4/93.1	
*Lb. paracasei* CECT 4022				64.2/89.9	
*Lb. plantarum* CECT 220				31.1/64.6	
*Lb. plantarum* CECT 221				29.6/64.4	
*Lb. plantarum* CECT 222				72.6/64.8	
*Lb. plantarum* CECT 223				63.7/66.3	
*Lb. plantarum* CECT 748				30.1/58.4	
*Lb. plantarum* CECT 749				90.5/97.1	
*Lb. rhamnosus* CECT 278T				92.1/90.3	
*Lb. rhamnosus* CECT 288				86/95	
*Lb. salivarius* CECT 4062				71.4/87.7	
*Leuconostoc mesenteroides* CECT 215				-/52.4	
*Acinetobacter* sp. Neg1 ITEM 17016	Rotatory shaker 120 rpm, 28 °C, 6 days	MMP Medium	1 µg/mL	>70%	[51]
*Brevidobacterium casei* (RM101, DSM 20657, DSM 9657, DSM 20658), *B. linens* DSM 20425, *B. iodinum* DSM 20626, *B. epidermidis* DSM 20660	Rotatory shaker 150 rpm, 30 °C, 10 days	BSM Medium	40 µg/L	100	[52]
Pediococcus parvulus UTAD 111B,	30 °C, 7 days	MRS broth	1 µg/mL	72%	[53]
UTAD 168,				89%	
UTAD 333,				97%	
UTAD 334,				94%	
UTAD 335,				98%	
UTAD 473				100%	
Aspergillus strains M100120	With agitation at 3.8× *g*, 30 °C, 6 days	MEA Medium	10 µg/mL	99%	[57]
M30011				81%	
M10012				76%	
M4001				71%	
X6121				44.3%	
X1011				30%	
*Aspergillus oryzae*	30 °C, 72 h	PDA Medium	10 µg/mL	94%	[58]

Abbreviation: BSM—Basal Salts Medium; LB—Lauria-Bertani; MEA—Malt Extract Agar; MMP—Minimal Medium Peptone; MRS—De Man, Rogosa, and Sharpe; PDA—Potato Dextrose Agar; PM—Polytoma Medium.

In a study performed by Prasad et al., piglets were fed with feed supplemented with OTA and OTA amidohydrolase (OAH). The results showed that piglets receiving feed containing OAH exhibited significantly lower OTA concentrations in plasma and dried blood spots, as well as in the kidney, muscle, and liver, with reductions of up to 67% observed in the liver [59].

Biotransformation of OTA through its degradation has also been investigated in in vivo studies (Table 2). In the study performed by Madhyastha et al., hydrolysis of OTA by the microbial activity of digesta in the gastrointestinal tract of Sprague-Dawley male albino rats was evaluated. Obtained results showed that digesta collected from the stomach or small intestine did not degrade OTA; however, the percentage of hydrolysis was observed in the digesta samples obtained from the cecum (52.5%) and large intestine (54.5%) after 6 h. Furthermore, the authors conducted a similar study using a diet supplemented with an antibiotic. Results showed that digesta from the rats administered with neomycin sulfate, had elevated pH value in the large intestine (*p* < 0.01) and cecum (*p* < 0.0001) and showed significantly lower rate of OTA hydrolysis in cecum (91.3% in control vs. 11.9% in neomycin group) and large intestine (84.7% in control group vs. 5.8% in the neomycin group) after 12 h of incubation [60].

In the study conducted by Kiessling et al., the degradation of various mycotoxins was evaluated in the sheep’s intact rumen fluid, rumen protozoa, and rumen bacteria. OTA was added to the diet in two different concentrations—2 mg/kg for the first 4 days and 5 mg/kg for the next 2 days. The concentration of OTA was examined in the rumen fluid samples, half an hour or more after feeding. In the lower concentrations, OTA was not detected in the tested samples; however, after receiving higher concentrations of OTA, its level was 29 ng/mL after half an hour and 14 ng/mL 1 h after feeding. A noteworthy conclusion drawn by the authors was that bacteria within the rumen fluid exhibited minimal involvement in the disappearance of OTA, whereas the greatest activity was shown for protozoa—an important component of the sheep’s microbiota [61]. Similar results were obtained in the Müller et al. [62] and Xiao et al. [63] studies.

**Table 2:** The summary of the in vivo experiments presenting the degradation of OTA.

Mechanism	Animal	Source/Conditions	OTA Concentration	The Percentage of OTA Degradation	Reference
OTA ⟶ OTα	Sprague-Dawley male albino rats	Digesta from cecum and large intestine. Incubated at 37 °C, 6 h in a shaking water bath	20 µg OTA addition into 1 g of digesta sample	Cecum: 52.5% Large intestine: 54.5%	[60]
OTA ⟶ OTα	Sheep	Rumen fluid	2 ppm and 5 ppm. OTA was added to the diet for 4 days.	99–100%	[61]
OTA ⟶ OTα	Brown Swiss Cow	Rumen fluid. 39 °C, 8 h in a shaking water bath, in the dark with constant CO_2_ administration.	Pure OTA was added at zero time (equivalent to 200 µg/L rumen fluid)	~100%	[62]
OTA ⟶ OTα	Suffolk sheep	Urine	i.v.: 0.2 mg/kg of bw i.r.: 0.5 mg/kg of bw	i.v.: 2–4% i.r.: 90–99%	[64]
OTA ⟶ OTα after OAH supplementation	Weaning piglets	Plasma, DBS, kidney, liver, muscle, GIT (digesta content of stomach, jejunum, cecum, and colon)	OTA: 50 or 500 µg/kg of bw OAH: 50 or 500 µg/kg of bw	Plasma: 54–59% DBS: 50–53% Kidney: 52% (OAH500) Liver: 67% (OAH500) Muscle: 59% (OAH500) GIT (OAH500): Stomach: 67% Jejunum: 68% Cecum: 86% Colon: 93%	[59]

Abbreviation: BW—Body weight; DBS—Dried Blood Spots; GIT—Gastrointestinal tract; i.r.—intraruminal; OHA—OTA amidohydrolase; OTA—Ochratoxin A.

The second mechanism responsible for OTA biodetoxification is adsorption, a process in which OTA binds to microorganisms, thereby preventing its absorption by the host organism and promoting its elimination. Adsorption results from interactions between microorganisms and the mycotoxin, which occur through both physical and chemical processes. The initial step involves the adhesion of OTA to surface proteins located on the cell wall or chemical compounds like polysaccharides (e.g., β-glucan, mannan) or peptidoglycan. The efficiency of adsorption depends on various factors, including the thickness, structure, and integrity of the cell wall, as well as the overall morphology of the organism capable of binding OTA. Further step involves the formation of complexes between OTA and the microorganism [39]. Lactic acid bacteria (LAB) are one of the most extensively studied microorganisms in the context of mycotoxin adsorption. Exhibiting the typical structure of Gram-positive bacteria, their cell wall is composed of thick and rigid peptidoglycan layers and glycopolymers, such as polysaccharides, teichoic, and lipoteichoic acids. The efficiency of mycotoxin binding by the cell wall of various strains classified as LAB may result from the amino acid sequence of peptides present in the peptidoglycan, as well as from the presence of negatively charged functional groups commonly found in the bacterial cell wall [65,66,67].

Piotrowska conducted a study evaluating the ability of LAB species (*Lactobacillus plantarum*, *Lactobacillus brevis*, and *Lactobacillus sanfranciscensis*) to adsorb OTA. The strains were inoculated into MRS medium and PBS buffer, each supplemented with 1000 ng/mL of OTA, and tested in two forms: viable and thermally inactivated cells. After 24 h of incubation, viable LAB reduced OTA level by 16.9–35% in MRS medium and 14.8–26.4% in PBS. This finding is noteworthy, as the lack of nutritional factors in PBS inhibits bacterial growth, indicating that active metabolism is not required for OTA removal. To further verify this hypothesis, LAB strains were heat-treated, and the results showed an enhanced reduction efficiency, reaching 46.2–59.8%, thus supporting that adsorption is the main mechanism involved. The improved performance of thermally inactivated cells was likely associated with protein denaturation and increased pore formation, which exposed additional binding sites for OTA. To investigate the contribution of the bacterial cell wall to OTA binding, Piotrowska performed an experiment using spheroplasts, which are bacterial cells with a partially removed cell wall. These modified bacteria exhibited very low adsorption efficiency (3.9–5.6%), confirming that an intact cell wall is essential for the binding process. For potential applications, it is very important to assess the stability of the toxin-cell wall complex; thus, weakly bound OTA may be released under gastrointestinal conditions due to continuous washing of the bacterial biomass [68]. LABs were also tested by Luz et al., in which their hydrolytic and adsorbing features were evaluated [56], and their effectiveness was confirmed in similar studies [69,70,71].

In the study performed by El Khoury et al., seven actinobacterial strains (AT10, AT8, SN7, MS1, ML5, G10, and PT1) were used to evaluate their role in binding and adsorbing OTA. Results showed that all of the tested strains possessed the ability to bind OTA on their surfaces, and the highest efficiency was found for the SN7 strain with a 33.93% binding capacity after 1 h [72].

The available literature also includes studies demonstrating the ability of microorganisms to eliminate OTA; however, based on the reported results and experimental methods, it remains unclear whether OTA degradation occurs enzymatically, leading to the formation of breakdown products, or primarily through adsorption. The impact of microorganisms, including *Saccharomyces cerevisiae*, *Lactobacillus*, and *Bacillus* strains, on OTA degradation was evaluated by Böhm et al., who demonstrated that *S. cerevisiae* O11 degraded 38% of OTA, *L. bulgaricus* 259/2 and *L. helveticus* were able to remove OTA by 94% and 72%, respectively, and *Bacillus* strains were able to remove 68% (*B. lichniformis*) and 39% (*B. subtilis*) of OTA [73]. In the study by Petchkongkaew et al., 23 *Bacillus* spp. strains were evaluated for their ability to degrade OTA, of which 15 were capable of removing the toxin. The highest removal efficiency was observed for *Bacillus licheniformis* CM21, thus reaching 81% [74]. Škrinjar et al. showed that the growth of *Streptococcus salivarius* subsp. *thermophilus* in milk with OTA significantly decreased the OTA concentration. Similar ability was shown for *L. delbrueckii* subsp. *bulgaricus* [75].

Yeasts, particularly those belonging to the *Saccharomyces* genus, are also capable of adsorption. The structure and composition of their cell wall enable adhesion of OTA. According to Jouany et al., the mechanism of mycotoxin binding to yeast cells is primarily based on physicochemical adsorption onto the cell wall surface. The yeast cell wall is mainly composed of polysaccharides, including β-D-glucans and chitin in the inner layer, and mannoproteins in the outer layer. Among these components, β-D-glucans play a major role in the complexation of mycotoxins. The process is not covalent but rather mediated through non-covalent interactions, including hydrogen bonding, Van der Waals forces, and, to some extent, hydrophobic interactions. Importantly, adsorption is reversible, thus part of the bound toxin can be released from the yeast surface [76].

In the study conducted by Armando et al., four *S. cerevisiae* strains (RC008, RC009, RC012, and RC016) were evaluated to assess their efficiency in removing OTA from the in vitro environment. Results showed that all tested strains adsorbed some part of the OTA. The binding ability varied between the strains, and with the 1 µg mL^−1^ OTA, the percentage ranged from 46 to 74%. For 5 µg/mL: 16–39.2%, for 10 µg/mL: 14,5–58%, for 40 µg/mL: 17.9–39.2%, and for 100 µg/mL: 56,7–74,2%. Furthermore, strains exposed to gastrointestinal conditions enhanced their adsorption to OTA, thus showing not only promising results in the in vitro study, but also underlining their potential in future in vivo studies [77].

Similar observations were reported by Bejaoui et al., who evaluated the capacity of six *Saccharomyces* strains (five *S. cerevisiae* (LALVIN BM45; LALVIN Rhône 2226; UVAFERM 43; LALVIN Rhône 2323 and LALVIN Rhône 2056) and one strain of *S. bayanus* (LALVIN QA23)) to remove OTA from both synthetic and natural grape juice. The study demonstrated that *Saccharomyces* yeasts reduce OTA mainly through adsorption, as no degradation products were detected. Furthermore, the authors compared the performance of viable cells (VC), heat-treated cells (HC), and acid-treated cells (AC), showing that both HC and AC exhibited significantly higher adsorption capacity than VC, regardless of the tested medium. The application of high temperature or acid treatment may affect the integrity of the cell wall, where destabilization under such harsh conditions exposes additional binding sites available for OTA adsorption [78]. The ability of *S. cerevisiae* cell wall components to bind OTA was also demonstrated in the study by Piotrowska et al. [79].

The summary of the microorganism that possesses the ability to adsorb OTA is presented in Table 3.

## 4. Understanding the Interplay Between Ochratoxin A, the Gut, and the Microbiome

**Figure 1:**
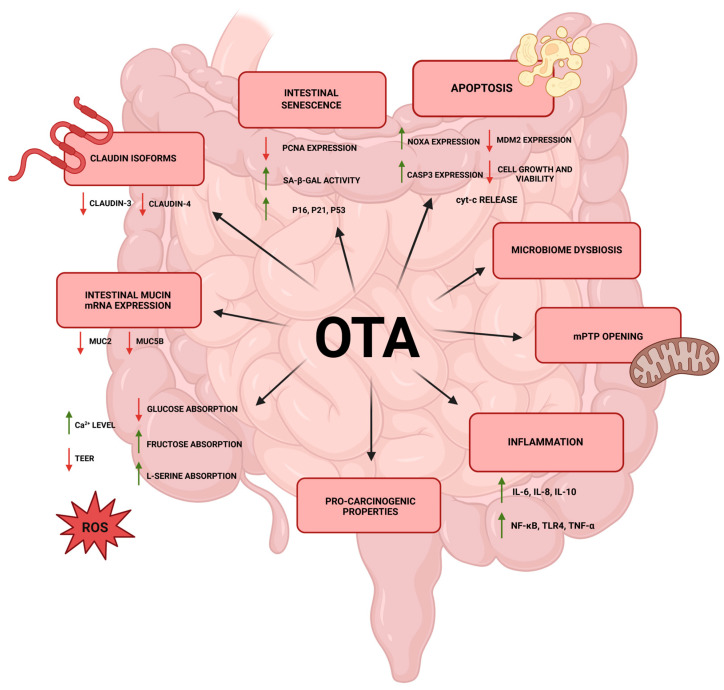
OTA toxicity in the intestines. Abbreviations: CASP3: caspase 3; cyt-c: cytochrome c; IL-6, IL-8, IL-10: interleukins; MDM2: murine double minute 2; mPTP: mitochondrial permeability transition pore; *MUC2*, *MUC5B*: mucin encoding genes; NF-κB: nuclear factor κ B; OTA: ochratoxin A; PCNA: proliferating cell nuclear antigen; P16, P21, P53: senescence-associated proteins; ROS: reactive oxygen species; SA-β-gal: senescence-associated β-galactosidase; TEER: transepithelial electrical resistance; TNF-α: tumor necrosis factor α; TLR4: toll-like receptor 4. Created in BioRender. Bijak, M. (2025) https://BioRender.com/skknjie (accessed on 27 August 2025).

Over the years, in vivo and in vitro studies have been conducted, indicating the toxic effects of OTA on the intestines (Figure 1). As summarized, tight junctions (TJ) in the intestinal epithelium are barriers for maintaining permeability since the TJ proteins prevent harmful substances from entering the body and maintain TJ integrity. However, OTA suppresses TJ proteins, including occludin, zonula occludens-1 (ZO-1), and claudin-1, in poultry, resulting in loss of TJ integrity and an increase in lipopolysaccharide (LPS) levels. Therefore, increased permeability leading to bacterial translocation can be observed [26].

An in vitro study on two human epithelial intestinal cell lines (HT-29-D4 and Caco-2-14) resulted in an inhibition of cellular growth and decreased transepithelial resistance. In addition, in HT-29-D4 cells, OTA caused a 60% decrease in sodium-dependent glucose absorption and an increase in absorption of fructose and L-serine [85]. Abassi et al. conducted studies on a human colon cancer cell line (HCT116), showing that exposure to OTA significantly reduced their clonogenic potential, which may be related to the induction of c-myc gene transcriptome expression. Moreover, OTA, even at very low concentrations, increased the migratory capacity of this cell line, suggesting its potential pro-carcinogenic properties in this experimental model [86]. Huang et al. reported that OTA leads to a significant reduction in intestinal Caco-2 and HT29-MTX cell viability, and transepithelial electrical resistance (TEER) values in a dose-dependent manner. Moreover, OTA significantly inhibited the expression of Mucin 2 (*MUC2*) and Mucin5B (*MUC5B*) genes, resulting in reduced synthesis and secretion of mucin—a key component of the intestinal mucus layer that plays an essential role in maintaining the integrity of the intestinal mucosa homeostasis [87]. Furthermore, OTA modulates the expression of *CYP450*, N-acetyltransferase 2 (*NAT2*), Cyclooxygenase-2 (*COX-2*), arachidonate 5-lipoxygenase (*LOX-5*) and *MRP2* genes in Caco-2 cells [88]. Studies on Caco-2 cells after 48 h of incubation with OTA showed that the toxin induces apoptosis, probably related to decreased expression of murine double minute 2 (MDM2) and increased expression of Noxa, and caspase 3 (CASP3) in a dose-dependent manner [89]. In an in vitro study, OTA was shown to alter epithelial permeability in Caco-2 cells through a mechanism involving the removal of a specific claudin isoform from the TJ. According to densitometric analysis of immunoblots, claudin-3 and claudin-4 signals were reduced by 87% and 72%, respectively, whereas no reduction in claudin-1 was observed. In addition, OTA did not affect the expression of ZO-1 and occluding proteins [90]. Similar results were obtained in the study performed by Romero et al., in which the reductions in claudin-3 and claudin-4 were observed [91]. Gao et al. reported that exposure to 4 µg/mL of OTA disrupted intestinal epithelial integrity in differentiated Caco-2 cells. Moreover, transcriptome and proteome analysis revealed down-regulation of genes and proteins linked to focal adhesion, adherens junctions, and gap junction pathways, indicating that OTA impairs gut barrier function [92].

Porcine epithelial cell line (IPEC-J2) was also studied for OTA-cytotoxicity. As a consequence of toxin administration, the researchers observed reactive oxygen species (ROS) generation, mitochondrial permeability transition pore (mPTP) opening, and cytochrome c (cyt-c) release. Moreover, OTA presence induced caspase-3 activation and cell apoptosis [28]. In addition, OTA exposure in IPEC-J2 cells resulted in a reduction in TEER, down-regulation in ZO-1 expression, redistribution of Occludin and ZO-1, increased Ca^2+^ level, and activation of myosin light chain kinase (MLCK). Consequently, OTA disrupts the TJ in the cell monolayer [93]. In an in vitro study, OTA altered the expression of 1678 genes in IPEC-J2 cells, with 782 being upregulated and 896 downregulated. Moreover, OTA led to a disruptive effect on the TJ protein ZO-1 and markedly induced the transcription of pro-inflammatory mediators, including interleukins (IL-6, IL-8, IL-10), nuclear factor κ B (NF-κB), toll-like receptor 4 (TLR4), and tumor necrosis factor α (TNF-α) [94].

Wang et al. performed an in vitro and in vivo study. As a result, in cultured intestinal cells, OTA exposure triggered calcium overload and oxidative stress, leading to inflammatory responses. This was accompanied by marked upregulation of senescence-associated proteins (P16, P21, P53), an increase in senescence-associated β-galactosidase (SA-β-gal) activity, and a reduction in proliferating cell nuclear antigen (PCNA) expression. Consistently, in vivo experiments demonstrated a significant enhancement of intestinal aging in mice exposed to OTA in comparison to controls. Therefore, OTA promotes intestinal senescence [95].

In the study performed by Nejdfors et al., gastrointestinal barrier function was investigated in human, rat, and pig tissues using Ussing diffusion chambers and standardized marker molecules. Across all species, permeability declined with increasing molecular weight. In humans, mannitol transport was greater in the small intestine than in the colon, whereas in rats it peaked in the ileum. Rats also showed higher FITC-dextran 4400 permeability than humans and pigs, while larger macromolecules crossed poorly in all cases. Overall, permeability patterns in pigs resembled those in humans more closely than in rats, suggesting limited applicability of rat data to human physiology [96].

During the necropsy, the researchers observed intestinal changes in OTA-fed pigs. As described, bile-stained fluid and gas caused distension of individual intestinal parts, such as the jejunum, ileum, spiral colon, and rectum. In addition, a thin and atonic intestinal wall was noted, as well as hemorrhages and a fibronecrotic membrane focally covering the mucosa in the spiral colon [97]. Another study performed by Solcan et al. resulted in the chickens’ intestinal mucosa changes post-OTA administration in three different concentrations (1 µg/kg bw/day, 20 µg/kg bw/day, and 50 µg/kg bw/day). OTA-cytotoxicity was observed after one week in groups fed with higher concentrations and after four weeks when fed with the lowest concentration. Additionally, both higher concentrations led to an increase in the diameter of jejunal intestinal villi, which was visible after 14 days of exposure to the toxin. Moreover, a time- and dose-dependent increase in their number was observed. The researchers observed necrotic areas only in the chickens that received the highest dose of OTA, while ochratoxin administration also affected the concentration of intraepithelial lymphocytes (T Cell Receptor 1 (TCR1), TCR2, Cluster of Differentiation 4+ (CD4+), CD8+) in the duodenum, jejunum, and ileocecal junction, high numbers of CD4+ and CD8+ lymphocytes were observed in the lamina propria [98]. In an in vivo study on broiler chickens exposed to both OTA and *Eimeria tenella* oocysts, the researchers demonstrated that the toxin exacerbated the pathological effects of *E. tenella* due to the immunosuppressive properties of OTA. Histopathological examination of the caecum revealed severe hemorrhage, numerous second-generation schizonts, mature merozoites, and developing oocysts [99]. In OTA-fed broiler chickens, histopathological changes were observed, as the examination of the intestinal mucosa revealed pronounced structural alterations, including villous thinning and basal enlargement, crypt hyperplasia with irregular morphology, as well as villous blunting and denudation. Biochemical analysis further demonstrated a reduction in mucosal lipid levels, such as phosphatidylcholine (PC) and phosphatidylethanolamine (PE) by 55.81% and 56.66%, respectively, and an increase in another mucosal lipid, such as phosphatidylserine by 32.91% [100]. In vivo studies in BALB/c mice showed that OTA disrupts intestinal barrier integrity by increasing apoptosis and reducing TJ protein expression. Transcriptome analysis revealed that OTA modulates key genes, including Dual-Specificity Phosphatase 9 (*DUSP9*), Phospholipase A2 group IID (*PLA2G2D*), and Serine/threonine-protein kinase PAK 6 (*PAK6*) via specific microRNA and long non-coding RNAs, implicating mitogen-activated protein kinase (MAPK) and rat sarcoma (Ras) signaling pathways in the observed intestinal damage [101].

Peng et al. conducted a study in which duck embryos injected with OTA exhibited disrupted jejunal TJ and increased secretion of inflammatory cytokines via activation of the TLR4 signaling pathway. Moreover, the researchers observed damage in the intestinal barrier manifested by shortened villi, crypt hyperplasia, disrupted intestinal TJ, and increased levels of LPS in the jejunum. Additionally, OTA caused microbiota dysbiosis and consequently led to increased *Bacteroides*, *Megamonas*, *Fournierella*, and decreased *Alistipes* and *Weissella* abundance [102]. In the in vivo study, following the administration of low doses of OTA, a decrease in microbial diversity and *Firmicutes* abundance, along with an increase in *Bacteroidetes* abundance at the phylum level, was observed in the fecal microbiota of mice. Moreover, the researchers demonstrated significant alteration among unclassified *Bacteroidales*, *Porphyromonadaceae*, unclassified *Cyanobacteria*, *Streptococcaceae*, *Enterobacteriaceae*, and *Ruminococcaceae* [103]. Guo et al. performed a study on male F344 rats administered with OTA in doses of 0, 70, or 210 μg/kg body weight. The results indicated that OTA treatment decreased the gut microbiota diversity of the gut microbiota, and a significant increase in *Lactobacillus*. Moreover, after 16S rRNA and shotgun sequencing, changes in signal transduction, carbohydrate transport, transposase, amino acid transport system, and mismatch repair were observed. In addition, isolated *Lactobacillus* species from fecal samples were compared to *Lactobacillus plantarum* strain PFK2, resulting in 99.8% 16S rRNA similarity. The researchers consider *Lactobacillus* as a key genus in in vivo OTA detoxification due to its ability to absorb OTA, excluding degradation [104].

The gut microbiota has been shown to modulate intestinal homeostasis through epigenetic mechanisms. Studied in germ-free and conventionally raised mice demonstrated that commensal microbiota induce localized, Ten-Eleven Translocation dioxygenases (TET2/3)-dependent DNA methylation changes at regulatory elements, activating key genes required for intestinal integrity. During inflammation, these epigenetic modifications alter gene expression programs associated with colitis and colon cancer, highlighting the critical role of microbiota-driven epigenetic programming in maintaining intestinal homeostasis [105]. In two Dutch population-based cohorts, blood DNA methylation profiles were associated with the abundance of the genus *Eggerthella*. The microbiome also appeared to mediate the effects of environmental factors on gene methylation, highlighting its potential role as an epigenetic regulator in humans [106]. Studies in rats have shown that OTA exposure leads to specific epigenetic alterations, including changes in promoter-region DNA methylation and gene expression. Key genes affected by OTA include GEN1 Holliday Junction 5′ Flap Endonuclease (*Gen1*), Annexin A3 (*Anxa3*), Cyclin-dependent kinase inhibitor 1A (*Cdkn1a*), and Oncostatin M (*Osm*), which play roles in DNA repair, cell proliferation, and senescence-associated pathways. Moreover, downregulation of *Gen1* and upregulation of *Anxa3* and *Osm* may impair DNA double-strand break repair while enhancing cell proliferation, exacerbating chromosomal instability [107]. Histone H3 crotonylation, a post-translational modification abundant in intestinal epithelial cells, is dynamically regulated during the cell cycle and controlled by class I HDACs. Microbiota-derived metabolites, such as short-chain fatty acids, modulate crotonylation, and depletion of gut microbiota alters global crotonylation patterns, linking microbial composition to host chromatin regulation [108]. In human kidney epithelial cells, OTA induces sustained mitotic arrest, aberrant chromosome condensation, and premature sister chromatid separation. These effects are associated with altered phosphorylation and acetylation of core histones, and OTA has been shown to inhibit histone acetyltransferase (HAT) activity in a concentration-dependent manner [109]. The gut microbiota impacts epigenetic mechanisms, including DNA methylation and histone modification. Similarly, OTA disrupts DNA methylation and histone acetylation, altering gene expression related to cell cycle, DNA repair, and senescence. These parallels suggest that OTA may interfere with microbiota-driven epigenetic regulation, highlighting the need for studies on their combined impact on intestinal barrier function and host health.

Although this review focuses on OTA toxicity in the intestine and its microbiome, its effects may extend to other organs. The gut microbiota influences host physiology through the gut-liver [110], gut-kidney [111], and gut–brain [112] axes. Wang et al. observed changes induced by OTA, including alterations in intestinal microbiota composition and structure, resulting in an increased amount of *Bacteroides*. Consequently, these bacteria led to LPS accumulation and inflammation in the liver due to activation of the liver TLR4/MyD88 signaling pathway [113]. In another study, Ma et al. reported OTA altered tryptophan metabolism, decreasing nicotinuric acid levels in the intestinal tract, which was negatively correlated with *Bacteroides plebeius*, and increasing indole-3-acetamide levels. These metabolic disturbances contributed to gut microbiota dysbiosis and resulted in hepatic NAD+ and ATP depletion. The subsequent AMP-activated protein kinase (AMPK) signaling activation and suppression of phosphorylated mechanistic target of rapamycin (mTOR) pathway indicate that OTA induces an energy-deficient state in the liver [114]. OTA induces kidney injury in animals, and the intestinal microbiota plays a key role in mediating this effect via the gut-kidney axis. Dysbiosis and altered microbial metabolites contribute to inflammation and renal damage, highlighting the importance of microbiota in OTA nephrotoxicity [115]. Furthermore, OTA induces hippocampal neurotoxicity via oxidative stress, inflammation, and mitochondrial dysfunction. Moreover, evidence indicates that these effects are linked to the gut–brain axis, as OTA alters gut microbiota composition and reduces short-chain fatty acid (SCFA) production, contributing to neuroinflammation and impaired synaptic function [116].

As noted, microbiome disruptions can lead to serious changes in the body, as they have been linked to the occurrence of cancer. This is why the term oncobiome was coined [117]. Moreover, the process of carcinogenesis does not require large changes in the microbiome, because even small ones can lead to deterioration of health and cause serious diseases. Additionally, the density of microorganisms is important in the process of cancer induction, which may affect the incidence of its occurrence depending on the characteristics of the microbiome in individual organs [32]. It is also important that disruption of the intestinal microbiota and increased LPS production may be the result of an existing cancer, which, in turn, additionally enhances carcinogenesis, as in the case of hepatocellular carcinoma (HCC) [118]. Furthermore, TLR4 signaling may be crucial as it links gut dysbiosis to HCC progression [119]. Many bacteria, in the case of microbiome disturbances, are associated with the formation of cancer, including colorectal cancer (CRC), gastric cancer, esophageal cancer, breast cancer, prostate cancer, lung cancer, melanoma, and pancreatic cancer [120]. CRC has been linked to *Escherichia coli* and *Bacteroides fragilis*, causing ROS generation and DNA damage in colonic epithelium. In addition, metagenomic studies have revealed increased abundance of *Fusobacterium nucleatum* in the tumor microenvironment. Consequently, passenger bacteria promote further inflammation through myeloid cell invasion and may contribute to the progression of CRC from early to more advanced stages [121]. As noted, microbiota is associated with local recurrence and metastasis, due to tumor cells shedding within the GIT, which are then influenced by the microbiome [122]. Additionally, cathepsin K secretion by *E. coli* and increased LPS levels increase metastasis. Furthermore, cathepsin K secretion could bind to TLR4, resulting in M2 polarization of tumor-associated macrophages (TAMs) via an mTOR-dependent pathway [123].

**Figure 2:**
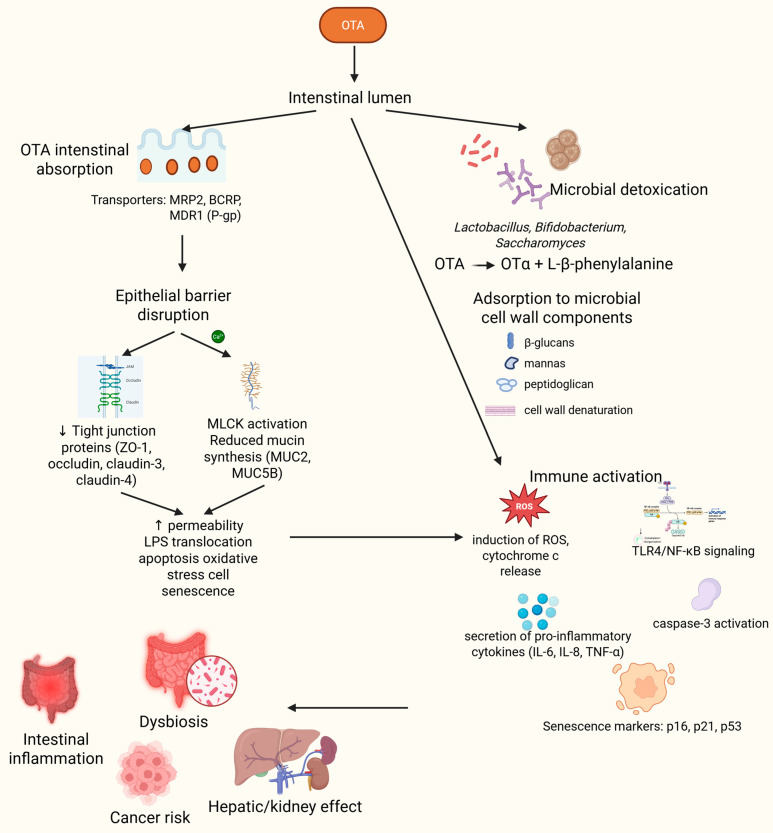
Mechanistic diagram of ochratoxin A (OTA) interactions within the intestine. After ingestion, OTA reaches the intestinal lumen, where it follows several parallel pathways. Intestinal absorption occurs through enterocytes and is modulated by efflux transporters such as MRP2, BCRP, and MDR1 (P-gp). Once absorbed, OTA binds strongly to serum albumin and undergoes enterohepatic recirculation, contributing to its systemic distribution. Microbial detoxification takes place via enzymatic hydrolysis by *Lactobacillus*, *Bifidobacterium*, and *Saccharomyces* species, converting OTA into the non-toxic metabolite ochratoxin α (OTα) and L-β-phenylalanine. In parallel, OTA can be adsorbed to microbial cell wall components (β-glucans, mannans, peptidoglycans), a process enhanced by cell wall denaturation. Epithelial barrier disruption is induced by OTA via downregulation of tight junction proteins (ZO-1, occludin, claudin-3/-4), activation of MLCK, resulting in increased intestinal permeability, LPS translocation, apoptosis, oxidative stress, and cellular senescence. Immune activation involves induction of ROS, cytochrome c release, caspase-3 activation, and upregulation of inflammatory signaling pathways (TLR4/NF-κB), leading to secretion of cytokines such as IL-6, IL-8, and TNF-α. (v) These mechanisms culminate in systemic outcomes, including intestinal inflammation, microbiota dysbiosis, hepatic and renal injury via the gut–liver/kidney axis, and potential cancer risk through chronic inflammation and oxidative DNA damage. Abbreviations: BCRP: breast cancer resistance protein; IL: interleukin; LPS: lipopolysaccharide; MDR1: Multidrug Resistance Protein 1; MLCK: myosin light chain kinase; MRP2: multidrug resistance-associated protein 2; NF-κB: nuclear factor kappa B; OTA: ochratoxin A; OTα: ochratoxin α; P-gp: glycoprotein P; ROS: reactive oxygen species; TLR4: toll-like receptor 4; TNF-α: tumor necrosis factor α; ZO-1: zonula occludens-1. Created in BioRender. Bijak, M. (2025) https://biorender.com/20neuth (accessed on 24 September 2025).

Extensive research on OTA has been conducted using in vitro models of human and animal cells, revealing its effects on intestinal barrier integrity, apoptosis, and gene expression. In vivo studies in animals have corroborated these findings, demonstrating OTA-induced intestinal toxicity. However, most evidence on OTA-gut microbiota interactions comes from animal models, and although human cohort studies provide initial indications of health risks associated with OTA exposure, the specific effects of chronic OTA intake on intestinal barrier integrity and gut microbiome remain insufficiently explored. This underscores the need for further research in humans to account for interspecies differences in microbiome composition, diet, and exposure levels, and to clarify the mechanistic links between OTA, gut microbiota, and host health. A schematic overview of these intestinal mechanisms is provided in Figure 2.

## 5. The Microbiome as a Determinant of Resistance to Toxins

**Figure 3:**
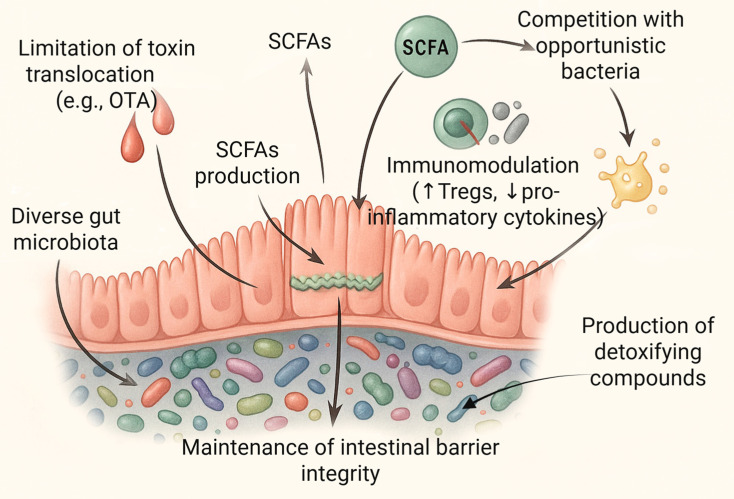
Mechanisms of microbiome-mediated protection against toxins. Abbreviations: OTA: ochratoxin A; SCFAs: short-chain fatty acids; Tregs: regulatory T cells. Created in BioRender. Bijak, M. (2025) https://BioRender.com/3wxgi69 (accessed on 27 August 2025).

The diversity of the gut microbiome, understood both as species richness and evenness, is recognized as one of the key indicators of intestinal ecosystem health. In the context of exposure to OTA, a high level of microbiome diversity may serve as a natural protective barrier, not necessarily through the direct degradation of the toxin, but via a range of indirect mechanisms associated with the maintenance of host homeostasis (Figure 3) [124,125].

One of the principal protective mechanisms linked to a diverse microbiome is the support of intestinal barrier integrity. Numerous commensal bacteria, including *Lactobacillus*, *Bifidobacterium*, *Akkermansia muciniphila*, and *Faecalibacterium prausnitzii*, synthesize metabolites such asSCFAs, primarily butyrate, which influence the expression of tight junction proteins and enhance the structure of the intestinal epithelium [126,127,128]. As a result, the translocation of harmful substances from the intestinal lumen into systemic circulation is limited, including that of OTA [129]. SCFAs also play a significant immunomodulatory role. They influence T lymphocyte activity, promoting the differentiation of regulatory T cells (Tregs) and attenuating inflammatory responses, which may otherwise be exacerbated in the presence of OTA and lead to secondary tissue damage [130,131]. Commensal microorganisms also compete for ecological niches and nutrients with opportunistic bacteria, thereby limiting the colonization of the gut by potentially pro-inflammatory microbes, whose presence may exacerbate the toxic effects of OTA [132,133].

A highly diverse microbiome also promotes the production of bioactive compounds with protective effects, including detoxifying enzymes, antioxidant molecules, and toxin-binding substances, even though these may not always directly modify the OTA structure [134,135]. Animal model studies have demonstrated that individuals with a richer microbiome exhibit less kidney and liver damage following OTA exposure compared to those with a depleted or artificially altered gut microbiota [103,113]. These correlations are also observed in human populations—individuals with greater gut microbiome diversity appear to be less susceptible to the toxic effects of certain xenobiotics, including mycotoxins [136,137].

Importantly, high microbiome diversity also ensures so-called functional redundancy, meaning the ability of different species to perform similar physiological functions. This implies that even in the absence of specific strains capable of directly degrading OTA, a protective effect may still be maintained through the activity of other microorganisms that support the intestinal barrier and immune system in analogous ways [138]. For this reason, interventions aimed at increasing microbiome diversity—such as diets rich in prebiotic fiber, consumption of fermented foods, or the reduction in unnecessary antibiotic use—may represent a promising strategy to enhance the body’s resistance to the toxic effects of OTA [139,140,141].

### 5.1. Dysbiosis and Its Implications for Susceptibility to OTA

Intestinal dysbiosis—defined as a disruption of microbiome homeostasis characterized by reduced microbial diversity, imbalanced community structure, and an increased presence of opportunistic microorganisms—is strongly associated with heightened susceptibility to environmental toxins, including mycotoxins such as OTA [124,142,143]. Under physiological (eubiotic) conditions, the gut microbiota plays a central role in maintaining intestinal barrier integrity, modulating immune responses, and supporting host detoxification pathways. When this balance is disturbed, these protective functions are impaired, leading to a cascade of pathophysiological events that may enhance the toxic effects of OTA [144].

One of the key mechanisms linking dysbiosis to OTA vulnerability is the compromised integrity of the intestinal epithelial barrier. Numerous studies have shown that microbiota disturbances contribute to increased intestinal permeability—a condition commonly referred to as “leaky gut”—which facilitates the translocation of OTA from the intestinal lumen into systemic circulation [145,146,147]. This is especially problematic under chronic exposure, where the intestinal barrier fails to effectively limit toxin absorption. Moreover, OTA itself exerts cytotoxic effects on epithelial cells and TJ proteins, and in the context of dysbiosis, this damage is amplified, creating a vicious cycle of barrier degradation and inflammation [103,148]. Recent preclinical studies have demonstrated that nutritional interventions such as astaxanthin supplementation can mitigate OTA-induced intestinal injury by modulating the composition of the gut microbiota and restoring tight junction integrity. In murine models, these effects were accompanied by an increase in goblet cell numbers, normalization of SCFA production, and suppression of the TLR4/MyD88/NF-κB signaling pathway [149].

Dysbiosis also leads to immune dysregulation, contributing to exaggerated or chronic inflammatory responses. In eubiotic conditions, the gut microbiota promotes the development of tolerogenic immune cell populations, such as Tregs, and suppresses excessive pro-inflammatory signaling. Conversely, dysbiosis often results in the overgrowth of potentially pathogenic bacteria, particularly *Proteobacteria* and members of the *Enterobacteriaceae* family—microbial signatures commonly associated with inflammatory and metabolic diseases [150,151,152]. These taxa stimulate the production of pro-inflammatory cytokines (e.g., IL-6, TNF-α, IL-1β), amplify immune responses to toxins, and may exacerbate tissue damage in OTA target organs such as the kidneys and liver [151,152,153].

Beyond intestinal and immunological effects, dysbiosis exerts systemic metabolic consequences through the gut–liver axis, including alterations in the expression of host detoxification enzymes and transport proteins. In particular, hepatic phase I and II detoxification enzymes such as CYP450 (CYP3A4, CYP2E1) are regulated by microbiota-derived metabolites (e.g., SCFAs, bile acids, indoles) through nuclear receptors like PXR (Nuclear receptor subfamily 1 group I member 2), CAR (Nuclear receptor subfamily 1 group I member 3), and transcription factors like AHR (Aryl hydrocarbon receptor). Dysbiosis may impair these signaling pathways, reducing the host’s capacity to metabolize and eliminate OTA efficiently [154,155]. Additionally, dysbiosis alters the expression and activity of intestinal and hepatic ATP-binding cassette (ABC) transporters such as glycoprotein P (P-gp) coded by the ABCB1 (ATP Binding Cassette Subfamily B member 1) gene, and MRP2 coded by the ABCC2 (ATP Binding Cassette Subfamily C member 2) gene, which are responsible for active efflux of OTA into bile or the intestinal lumen. Decreased expression or function of these transporters under dysbiotic conditions has been observed in chronic inflammation and disease states, leading to toxin accumulation and increased systemic bioavailability [156,157].

Crucially, dysbiosis also reduces the production of beneficial microbial metabolites, particularly SCFAs such as butyrate, acetate, and propionate. These metabolites are essential not only for maintaining epithelial integrity and immune tolerance, but also for regulating host cellular pathways related to oxidative stress and detoxification. Butyrate, for instance, activates AMPK and peroxisome proliferator-activated receptor gamma (PPAR-γ), and inhibits histone deacetylases (HDACs), thereby modulating transcriptional responses to toxic stressors [158]. The depletion of butyrate-producing taxa such as *Faecalibacterium prausnitzii*, *Roseburia*, and *Butyricicoccus* compromises these protective mechanisms and may contribute to increased OTA toxicity.

Experimental data strongly support these mechanisms. Mice subjected to antibiotic-induced dysbiosis exhibit higher plasma OTA concentrations, increased oxidative stress markers, histological damage in renal and hepatic tissues, and altered detoxification gene expression profiles [159]. In vitro models show that epithelial monolayers exposed to OTA in SCFA-depleted or pathogen-colonized environments suffer more severe cellular injury and loss of barrier function [139]. Recent studies also suggest that dietary strategies aimed at restoring microbial balance can alleviate OTA toxicity. In OTA-exposed ducks, curcumin supplementation reduced liver oxidative injury, improved microbial richness, and increased the abundance of SCFA-producing bacteria such as *Butyricicoccus* and *Blautia*. These changes were associated with enhanced antioxidant enzyme activity and improved lipid metabolism, reinforcing the central role of the microbiota in OTA toxicodynamics [160].

Finally, it is important to note that dysbiosis can arise from multiple non-antibiotic factors, including low-fiber diets, chronic stress, infections, inflammatory bowel disease, and environmental exposures such as pollutants and mycotoxin-contaminated foods. These conditions often co-occur in populations at high risk of OTA exposure, especially in low-resource settings where dietary diversity is limited [161,162].

To consolidate the insights discussed in this section, Table 4 outlines the main mechanisms through which gut dysbiosis may facilitate OTA toxicity.

### 5.2. Individual Microbiome Profiles and Variability in Toxin Susceptibility

In recent years, there has been a growing interest in characterizing individual differences in gut microbiome composition—not only in terms of taxonomic profiles, but also with regard to functional potential [163,164,165]. In the context of dietary toxicology, particularly in relation to OTA, a key emerging question is whether certain microbial profiles—referred to as microbiotypes—can predispose individuals to higher or lower susceptibility to the adverse effects of this mycotoxin.

Microbiotypes are defined as relatively stable configurations of dominant bacterial groups that reflect not just taxonomic structure but also broader ecological interactions between microbes and the host. Population-based studies have identified recurrent enterotypes dominated by *Bacteroides*, *Prevotella*, or *Ruminococcus*, which differ in multiple physiological attributes, including the production of SCFAs, fiber utilization efficiency, and pro-inflammatory potential [166,167,168]. While the application of this concept to OTA susceptibility is still in its early stages, preliminary evidence suggests that gut microbial composition may have prognostic value in estimating toxicological risk [39]. Table 5 summarizes hypothetical associations between microbiome configurations and susceptibility to OTA, based on their functional traits and current preclinical evidence.

In animal studies, individuals with differing microbial profiles have shown markedly variable responses to identical OTA doses, even under controlled environmental and dietary conditions. These differences were observed not only in terms of kidney tissue damage but also with respect to immunological responses and toxin metabolism [113,170].

Growing emphasis is now being placed on the functional characterization of the microbiome using metatranscriptomic, metaproteomic, and metabolomic approaches. These analyses enable the identification of specific metabolic pathways or gene expression signatures that may modulate OTA susceptibility. For instance, the expression of microbial genes involved in phenol metabolism, antioxidant biosynthesis, or host signaling modulation (e.g., Nuclear factor erythroid 2-related factor 2 (Nrf2), NF-κB, PXR pathways) may be more informative than the mere presence or absence of particular bacterial species [169,171,172]. Such functional biomarkers could help identify microbial configurations that either promote detoxification or exacerbate toxicokinetic processes.

The potential use of microbiotypes as predictive biomarkers in toxicology is an area of active investigation [173,174]. In the future, microbiome profiling could support clinical or nutritional decision-making, particularly for individuals with chronic dietary exposure to mycotoxins. Such strategies could enable the stratification of at-risk populations, guide personalized probiotic or dietary interventions, and refine the assessment of detoxification efficacy in individual patients [175,176,177].

Additionally, emerging tools such as machine learning and systems biology modeling are being employed to integrate microbiome, metabolome, and host-response data into predictive frameworks. These tools may eventually allow for the development of individualized “toxicity risk scores” based on microbiome data. This approach aligns with the broader vision of precision toxicology, in which host–microbiota interactions are explicitly considered in risk assessment and mitigation strategies [178,179].

## 6. Conclusions

Human exposure to OTA has been consistently demonstrated in biomonitoring studies across different regions of the world, indicating that this mycotoxin is a relevant public health concern rather than solely an experimental toxicant. OTA has been detected in urine and plasma samples of adults worldwide, suggesting the risk of chronic background exposure through diet [180,181,182,183,184]. In the study performed by Carballo et al., 40 male and female urine samples were collected from the citizens of Valencia (Spain) and were examined for the presence of deoxynivalenol, OTA, and zaeralenone. The results showed that 72.5% of examined samples were contaminated by at least one mycotoxin [185]. In the study conducted by Arce-Lopez et al., 19 various compounds, including mycotoxins and their metabolites, were assessed in plasma samples from 438 healthy donors from Navarra (Spain). OTA was found in 97.3% of the studied cases, with concentrations ranging from 0.4 to 45.7 ng/mL [186]. Comparable biomonitoring surveys performed worldwide confirmed that OTA is consistently present in blood and urine samples, thus highlighting its global relevance. Special attention should be given to the potential exposure of highly susceptible populations. A study published in 2024 demonstrated that OTA was detectable in 62.5% of breast milk samples and 51.4% of urine samples collected from nursing mothers in Bangladesh, thus raising concern for early-life exposure [187].

Evaluating whether exposure to a specific agent, like mycotoxin, can elevate the risk of developing a certain health condition has been undertaken by authoritative institutions like the European Food Safety Authority (EFSA) [188]. OTA has been classified as a renal carcinogen in particular animal and in vitro models and is known to induce nephrotoxicity. In humans, however, identifying specific hazards has proven more challenging. Exposure to OTA has been linked to several adverse renal outcomes, including Balkan Endemic Nephropathy (BEN), although the evidence is less definitive than that observed in laboratory animals. BEN, a chronic tubulointerstitial kidney disease with increased incidence of urothelial and renal carcinomas, is characterized by a pattern of occurrence that is familial yet not genetically inherited, typically appearing after residents have lived in an endemic village for at least 15 years, and is associated with renal carcinomas [189]. Despite earlier suspicions regarding OTA, many studies showed that OTAs do not form a specific OTA-DNA adduct and should not be considered as an etiological factor involved in BEN [188,190,191,192]. Furthermore, besides nephrotoxicity, it should be noted that direct assessment of hepatotoxicity or carcinogenicity in humans is not feasible due to the ethical constraints; therefore, human studies and epidemiological observations are limited to in vitro experiments using human cell lines and/or animal models, which can provide mechanistic insight [2,193].

Microbial-based strategies represent a highly promising approach for the mitigation of OTA exposure, not only in the food and feed system but potentially within the GIT. Numerous in vitro and in vivo studies have demonstrated that probiotic strains, including *Lactobacillus*, *Pediococcus*, *Bifidobacterium*, and *Saccharomyces* species, can effectively reduce OTA bioavailability through two complementary mechanisms: enzymatic hydrolysis and/or adsorption onto cell wall components such as polysaccharides, peptidoglycans, and mannoproteins. In the study by Śliżewska et al., the use of probiotic bacteria and yeasts in the preparation of OTA-contaminated feed for chickens resulted in a 73% reduction in OTA concentration after 6 h [194]. In the study by Markowiak et al., the authors aimed to evaluate the detoxification potential of individual probiotic strains derived from poultry synbiotic formulations against OTA, and to assess the genotoxicity of fecal water in chickens after 42 days of OTA exposure. In this experiment, three different synbiotics were used, with compositions varying in the number of *Lactobacillus* spp. strains (ranging from three to five). Additionally, each synbiotic contained *S. cerevisiae* and 2% inulin. The results demonstrated that all examined LAB and yeast strains were capable of detoxifying OTA (*p* < 0.05). Moreover, synbiotics containing four or five LAB strains significantly reduced the genotoxicity of chicken fecal water after OTA exposure, suggesting that the use of synbiotics could be considered a preventive strategy against OTA contamination in poultry [129]. Similar results were obtained in the Śliżewska et al., study, which aimed to evaluate the effects of synbiotics on growth performance, intestinal microbiota composition, and enzymatic activity in turkeys fed OTA-contaminated feed compared to OTA-free turkeys. Results showed that supplementation with synbiotics enhanced the abundance of beneficial bacteria and decreased potential pathogenic populations in the GIT. Furthermore, synbiotics increased the activity of α-glucosidase and α-galactosidase while lowering the activity of potentially harmful fecal enzymes, including β-glucosidase, β-galactosidase, and β-glucuronidase [195]. Similar results were also obtained in other studies [196,197].

Recent advances have also highlighted the potential of postbiotics as modulators of OTA toxicity. Unlike probiotics, which rely on the metabolic activity of viable microorganisms, postbiotics consist of inactivated microbial cells or their metabolites that can exert beneficial biological effects without the risks associated with live organisms. In a recent study, Jiang et al. demonstrated that tropical postbiotics markedly alleviated OTA-induced dysbiosis and kidney injury in vivo. OTA exposure disrupted gut microbial balance, enriched opportunistic taxa such as *Alistipes*, and suppressed antioxidant signaling via the Nrf2/HO-1 pathway, thereby promoting oxidative stress, ferroptosis, and renal damage. Administration of postbiotics not only normalized gut microbial composition but also reduced the abundance of microbial genes linked to virulence and iron uptake, while restoring Nrf2/HO-1 activity and ROS scavenging capacity, ultimately mitigating chronic kidney injury. These findings suggest that postbiotics may represent a promising adjunct or alternative to probiotic interventions, offering stability, safety, and reproducible bioactivity. Moreover, their ability to modulate both microbial ecology and host antioxidant defenses underscores their translational potential in strategies aimed at reducing the health risks posed by OTA exposure [198].

The high application potential in OTA biodetoxification can also be attributed to Fecal microbiota transplantation (FMT). Although no empirical studies to date have specifically employed FMT as a therapy to mitigate OTA toxicity, it remains a promising avenue for future investigation. FMT is a next-generation intervention that mainly focuses on reshaping the gut microbiome by transferring a healthy microbiota from a donor to a recipient to restore microbial balance. This therapeutic approach has gained attention for its potential to treat various conditions with gut dysbiosis, including bacterial infections (i.e., *Clostridium difficile*), irritable bowel syndrome, Crohn’s disease, diabetes mellitus, and obesity [199]. By restoring a balanced gut microbiota, FMT may enhance the host’s ability to metabolize and detoxify OTA. The healthy microbiota could facilitate the biotransformation of OTA into less toxic metabolites, thereby reducing its systemic burden. Additionally, FMT could strengthen the intestinal barrier function and support the integrity of TJs, mitigate inflammation, and restore homeostasis, collectively counteracting the adverse effects of OTA-induced dysbiosis.

To sum up, human exposure to OTA is a global and persistent concern, as confirmed by biomonitoring studies that detect this mycotoxin in blood, urine, and even breast milk samples. Although the direct causal link between OTA and specific human diseases remains less conclusive compared to animal and in vitro models, its harmful effects emphasize the need for continuous surveillance and risk assessment, especially in the context of dietary exposure.

## Figures and Tables

**Table 3:** The summary of the microorganisms able to adsorb OTA.

Microorganism	Reaction Conditions	Medium	OTA Concentration	The Percentage of OTA Degradation	Reference
*Saccharomyces cerevisiae*: Malaga LOCK 0173	30 °C, 24 h, thermally inactivated cells	YPG medium White grape juice medium Blackcurrant juice medium	1 µg/mL	YPG medium: 35.4% White grape juice medium: 82.8% Blackcurrant juice medium: 10.7%	[80]
Syrena ŁOCK 0201				YPG medium: 21% White grape juice medium: 85.1% Blackcurrant juice medium: 65.2%	
Bakery BS				YPG Medium: 54.1% White grape juice medium: 64.4% Blackcurrant juice medium: 62.4%	
*Candida intermedia*	Rotary shaker 100 rpm, 25 °C, 48 h, in dark, immobilized yeast cells on calcium alginate beads	Grape juice	20 µg/kg	>80%	[81]
*Debaryomyces hansenii*	Rotary shaker 300 rpm, 28 °C, 24 h, pH 3	YMB medium	7 µg/mL	>98% After 5 min	[82]
*Saccharomyces cerevisiae*	12 °C, 90 days, viable cells	Red, rose, and white wine musts	0.01–4 µg/mL	Red: 88–90% Rose: 83–86% White: 73–76%	[83]
*Lactobacillus kefiri* KFLM3,	Aerobically, 25 °C, 24 h, viable cells	Milk	1 µg/mL	81%	[84]
*Kazachstania servazzi* KFGY7,				62%	
*Acetobacter syzygii* KFGM 1				50%	
*Lactobacillus plantarum* LOCK 0862	30 °C, 24 h, viable cells	MRS medium	(A) 1 mg s.m./mL (B) 5 s.m./mL	(A) 21.23% (B) 35.01%	[68]
*Lactobacillus brevis* LOCK 0845				(A) 14.64% (B) 20.53%	
*Lactobacillus sanfranciscensis* LOCK 0866				(A) 16.91% (B) 32%	
Actinobacterial strains:	1 h, viable cells	PBS solution	45.12 ng/mL		[72]
AT10				25.62%	
AT8				16.07%	
SN7				33.93%	
MS1				4.33%	
ML5				9.46%	
G10				16.28%	
PT1				24.85%	
*Saccharomyces cerevisiae*: RC008	37 °C, 24 h	YPD medium	(A) 1 µg/mL (B) 5 µg/mL (C) 10 µg/mL (D) 40 µg/mL (E) 100 µg/mL	(A) 46% (B) 16% (C) 14.5% (D) 17.9% (E) 56.7%	[77]
RC009				(A) 43% (B) 16% (C) 49.4% (D) 37.3% (E) 67.2%	
RC012				(A) 63% (B) 39.2% (C) 56.4% (D) 39.2% (E) 71.2%	
RC016				(A) 74% (B) 30.4% (C) 58% (D) 39.2% (E) 74.2%	
*Saccharomyces cerevisiae* LALVIN BM45 LALVIN Rhône 2226 UVAFERM 43 LALVIN Rhône 2323 LALVIN Rhône 2056 *S. bayanus* LALVIN QA23	30 °C, 2 h, viable cells	YPD medium SGM medium	2 µg/mL	YPG medium: 11–45% SGM medium: 1–35%	[78]
*Bifidobacterium bifidum* CECT 870T	37 °C, 24 h	MRS Medium	0.6 µg/mL	pH 3.5/pH 6.5 9.1/3.1%	[56]
*Bf. breve* CECT 4839T				4.2/1.4%	
*Lactobacillus bulgaricus* CECT 4005				16/1.6%	
*Lb. casei* CECT 4040				5.3/3.5%	
*Lb. casei* CECT 4045				6.2/2.2%	
*Lb. johnsonii* CECT 289				16.3/4%	
*Lb. paracasei* CECT 4022				9.4/3.3%	
*Lb. plantarum* CECT 220				2.1/7%	
*Lb. plantarum* CECT 221				1.1/4.8%	
*Lb. plantarum* CECT 222				4.6/5.4%	
*Lb. plantarum* CECT 223				1.2/1.2%	
*Lb. plantarum* CECT 748				3/5.6%	
*Lb. plantarum* CECT 749				5.3/1.7%	
*Lb. rhamnosus* CECT 278T				5.6/3.3%	
*Lb. rhamnosus* CECT 288				5.1/2.1%	
*Lb. salivarius* CECT 4062				16.1/4.4%	
*Leuconostoc mesenteroides* CECT 215				-/-	

Abbreviation: MRS—De Man, Rogosa, and Sharpe; SGM—Synthetic Grape Juice Medium; YMB—Yeast Mold Broth; YPG—Yeast Peptone Glucose.

**Table 4:** Pathophysiological consequences of gut dysbiosis that increase susceptibility to OTA toxicity.

Mechanism	Description	Reference
Increase in intestinal permeability	Loss of tight junction integrity facilitates OTA translocation	[145,146,147,149]
Decrease in SCFA production	Depletion of butyrate producers impairs barrier function and immune control	[103,139,158]
Increase in inflammation	Expansion of Proteobacteria, ↑ IL-6, TNF-α, leads to tissue sensitization	[150,151,152,153]
Decrease in detoxication enzymes	Disrupted signaling to CYP450s (e.g., via PXR) lowers OTA metabolism	[154,155,159]
Decrease in transporter activity	Decreased P-gp/MRP2 reduces OTA excretion	[156,157]
Increase in oxidative stress	Lower antioxidant capacity in dysbiotic microbiota increases damage	[159,160]

Abbreviation: CYP450—cytochrome P450; IL-6—Interleukin 6; MRP2—multidrug resistance-associated protein 2; OTA—Ochratoxin A; P-gp—glycoprotein P; PXR—nuclear receptor subfamily 1 group I member 2; SCFA—short-chain fatty acid; TNF-α—tumor necrosis factor α.

**Table 5:** Potential mechanisms linking microbiome configurations to OTA susceptibility.

Microbiotype	Dominant Genus	Functional Features	Possible Effect on OTA Toxicity	Level of Evidence	References
Bacteroides	Bacteroides	Low SCFA, bile acid metabolism	Possibly higher risk	Moderate (indirect)	[167,168,169]
Prevotella	Prevotella	High SCFA, fiber fermentation	Possibly protective	Moderate (indirect)	[161,167,168]
Dysbiosis/increse of Proteobacteria level	Proteobacteria	Inflammation, decrease in detox enzymes	Higher risk	Strong (preclinical)	[150,151,153]

Abbreviation: OTA—Ochratoxin A; SCFA—short-chain fatty acid.

## Abbreviations

The following abbreviations are used in this manuscript:

ABC	ATP-binding cassette
ABCB1	ATP binding cassette subfamily B member 1
ABCC2	ATP binding cassette subfamily C member 2
AC	acid-treated cells
AFB1	aflatoxin B1
AHR	aryl hydrocarbon receptor
AMPK	AMP-activated protein kinase
Anxa3	Annexin A3
ATP	adenosine triphosphate
BCRP	breast cancer resistance protein
BEN	Balkan Endemic Nephropathy
BSM	basal salts medium
CAR	nuclear receptor subfamily 1 group I member 3
CASP3	caspase 3
CD	Cluster of Differentiation
Cdkn1a	Cyclin-dependent kinase inhibitor 1A
COX-2	Cyclooxygenase-2
CP	carboxypeptidase
CRC	colorectal cancer
CYP450	cytochrome P450
cyt-c	cytochrome c
DOX	deoxynivalenol
DUSP9	Dual-Specificity Phosphatase 9
EFSA	European Food Safety Authority
FBs	fumonisins
FMT	Fecal microbiota transplantation
Gen1	GEN1 Holliday Junction 5′ Flap Endonuclease
GIT	gastrointestinal tract
HAT	histone acetyltransferase
HC	heat-treated cells
HCC	hepatocellular carcinoma
HCT116	human colon cancer cell line 116
HDACs	histone deacetylases
IL	interleukin
IPEC-J2	intestinal porcine epithelial cells-jejunum 2
LAB	lactic acid bacteria
LB	Lauria-Bertani
LOX-5	arachidonate 5-lipoxygenase
LPS	lipopolysaccharide
MAPK	mitogen-activated protein kinase
MDM2	murine double minute 2
MDR1	multidrug resistance protein 1
MEA	malt extract agar
MLCK	myosin light chain kinase
MMP	minimal medium peptone
mPTP	mitochondrial permeability transition pore
MRP2	multidrug resistance-associated protein 2
MRS	De Man, Rogosa and Sharpe
mTOR	mechanistic target of rapamycin
MUC2	mucin 2
MUC5B	mucin 5B
NAD+	oxidized nicotinamide adenine dinucleotide
NAT2	N-acetyltransferase 2
NF-κB	nuclear factor kappa-light-chain enhancer of activated B cells
Nrf2	nuclear factor erythroid 2-related factor 2
OAH	ochratoxin amidohydrolase
Osm	Oncostatin M
OTA	ochratoxin A
OTα	ochratoxin α
PAK6	Serine/threonine-protein kinase PAK 6
PC	phosphatidylcholine
PCNA	proliferating cell nuclear antigen
PDA	potato dextrose agar
PE	phosphatidylethanolamine
P-gp	glycoprotein P
PLA2G2D	Phospholipase A2 group IID
PM	polytoma medium
PPAR-γ	peroxisome proliferator-activated receptor gamma
PXR	nuclear receptor subfamily 1 group I member 2
Ras	rat sarcoma
ROS	reactive oxygen species
SA-β-gal	senescence-associated β-galactosidase
SCFAs	short-chain fatty acids
SGM	synthetic grape juice medium
TAMs	tumor-associated macrophages
TCR	T cell receptor
TEER	transepithelial electrical resistance
TET	Ten-Eleven Translocation dioxygenases
TJ	tight junction
TLR4	toll-like receptor 4
TNF-α	tumor necrosis factor α
Tregs	regulatory T cells
VC	viable cells
YMB	yeast mold broth
YPG	yeast peptone glucose
ZO-1	zonula occludens-1
